# An (un)healthy social dilemma: a normative messaging field experiment with flu vaccinations

**DOI:** 10.1186/s13561-022-00385-9

**Published:** 2022-08-02

**Authors:** Irene Mussio, Angela C. M. de Oliveira

**Affiliations:** 1 Newcastle University Business School (Economics), 5 Barrack Road, Newcastle upon Tyne, NE1 4SE UK; 2 Department of Resource Economics, University of Massachusetts, 203 Stockbridge Hall, 80 Campus Center Way, Amherst, MA 01003 USA

**Keywords:** Influenza, Vaccination, Normative messaging, Public good, Joint product

## Abstract

**Background:**

Influenza seasons can be unpredictable and have the potential to rapidly affect populations, especially in crowded areas. Prior research suggests that normative messaging can be used to increase voluntary provision of public goods, such as the influenza vaccine. We extend the literature by examining the influence of normative messaging on the decision to get vaccinated against influenza.

**Methods:**

We conduct a field experiment in conjunction with University Health Services, targeting undergraduate students living on campus. We use four posters, randomized by living area clusters to advertise flu vaccination clinics during the Fall. The wording on the posters is varied to emphasize the individual benefits of the vaccine, the social benefits of the vaccine or both benefits together. We collect survey data for those vaccinated at the vaccination clinics, and for those not vaccinated via an online survey.

**Results:**

We find that any normative message increases the percentage of students getting the flu vaccine compared with no message. In terms of the likelihood of getting the flu vaccine, emphasizing both the individual and social benefits of vaccination has the largest increase in the vaccination rate (19–20 percentage point increase). However, flu vaccinations did not reach the herd immunity threshold (70% of students vaccinated).

**Conclusions:**

This study provides evidence that there is a pro-social component that is relevant in individual vaccination decisions which should be accounted for when designing vaccination campaigns. The results of this normative, pro-social messaging experiment could be extended to other at-risk communities where the number of background risks is much larger. This is especially relevant nowadays, as other seasonal vaccines are being rolled out and younger adults are the ones with the lowest uptake.

**Supplementary Information:**

The online version contains supplementary material available at 10.1186/s13561-022-00385-9.

## Introduction

Yearly seasonal influenza (flu) epidemics can seriously affect populations through rapid spreading of the disease, particularly in crowded areas such as schools or college residences [1]. Flu seasons can be unpredictable, and vaccination is one of the ways to prevent infection and related illnesses, as well as to reduce the severity of the symptoms and the likelihood of hospitalization if one catches the flu. The Centers for Disease Control (CDC) recommends the flu vaccine for everyone six months or older. Vaccination coverage in the United States, however, is far below the public policy objectives, as more than half of the population is typically unvaccinated (target is 70%). With the aim of increasing vaccination coverage, we analyze whether a flu vaccination campaign is successful at increasing flu vaccinations depending on the message transmitted to different groups of individuals.

Vaccines protect the individual who gets immunized and helps create a barrier for those who cannot get vaccinated, building herd immunity.^1^ Flu vaccines can therefore be considered a joint product, as vaccines have both individual and social benefits. However, when it comes to getting vaccinated, individuals have an incentive to free ride, avoiding the cost of the vaccine and indirectly benefiting from others getting vaccinated. Thus, herd immunity is a social dilemma, as vaccinations help the individual and others, but it is costly for someone to get the vaccine. One non-pecuniary mechanism which could influence vaccination decisions while highlighting the benefits of the vaccine is normative messaging [3, 4]. The present paper incorporates normative messages that appeal to these benefits (individual and social) into an established University campus-wide flu vaccination campaign. Normative messaging is done by incorporating social norms into the posters used to advertise the location and times of flu clinics on campus. We examine whether emphasizing each benefit of the vaccine (in terms of a reduction in risk) influences the decision to get vaccinated.

This paper contributes to the experimental vaccination literature by examining actual flu vaccination decisions as opposed to intention to vaccinate [5–7] We focus on individual demand-side decisions. This complements a significant body of experimental work related to the supply side of health markets and services, such as insurance markets [8] and physician behavior [9]. We gather data on both vaccinated and non-vaccinated individuals on campus to examine the influence of the campaign on student vaccination levels. We also contribute to the analysis of the influence of social norms on individual decision-making [10–13], as our experimental design allows us to expose large numbers of students to different normative messages. Understanding if reminders about benefits of the vaccine throughout the flu season can address part of the individual processes behind the decision to get vaccinated serve as an input for more effective and efficient interventions to tackle vaccine hesitancy [5, 6].

Our findings show that emphasizing both the individual and social benefits of vaccination has the largest increase in the vaccination rate, compared to emphasizing one benefit or when no benefit of the vaccine was highlighted (our baseline). This shows that there is synergy between both the self-interest and the pro-social or altruistic components of individual vaccination decisions. Our finding is also evidence that there is a pro-social, community component that is relevant in individual vaccination decisions which should be accounted for when designing vaccination campaigns by health services providers.

## Background

There are two main strands of literature relevant to our study: the influence of social norms on health-related behaviors and voluntary public goods provision in the case of vaccinations. We briefly discuss these literatures below.

### Influencing health-related behaviors

Many public health interventions attempt to influence the way people consciously think about their health behaviors. Inducing beneficial habits, such as exercising or quitting to smoke, by adjusting perceptions of social norms could significantly reduce health care provision costs [14, 15].

Normative influence is one avenue to affect behavior. Normative influence can be defined as a form of influence that uses perceived behavioral patterns as well as approval and disapproval of social norms to modify behavior [13]. Norms can be used to induce both individual and collective change, particularly when nudges focus on social comparisons [16, 17]. Norms are expected to affect behavior when salient at the time of the decision: that is, when peoples’ attention can be drawn to the norms [18]. Norms are also most influential when they are common to social groups, especially for health-related behaviors [19–21]. Normative nudges should increase welfare: anyone who is already behaving according to the norm used for the nudge will not be affected and those who are not will make decisions that improve their welfare if the nudge is successful in changing behaviors [16, 22]. In addition, health-based nudges have been shown to have high levels of acceptance, more so when there is high trust in the authorities [23, 24].

In the case of health-related behaviors, normative influence has been previously shown have short-run effects. These effects include increases in physical activity [25], healthier food consumption [14, 26] and reductions in alcohol consumption [27]. However, the decision to exercise, eat healthy food or drink alcohol are daily decisions with short-lived effects. The flu vaccine, in comparison, is a yearly one-time decision with longer-term effects (protection from the virus). Getting a flu vaccine is therefore a promising avenue for a normative-based intervention.

Normative influence can occur through social interactions and visual cues, such as signs, posters or electronic communications. Visual cues are particularly useful when the target audience is large and harder to reach and where interactions are repeated, such as hotel guests or college students. In the past, descriptive and injunctive messages have been used to incentivize different types of behavior, such as healthy food choices (descriptive norms provide information about how to act in a certain situation while injunctive norms refer to conducts that the majority of individuals approve or disapprove of) [28]. The studies on food choices finds that healthy descriptive messages result in healthier food choices compared to no message, an unhealthy descriptive or an injunctive message. Similar results have been found by using descriptive norm manipulations to analyze the choice between taking the elevator and using the stairs in university buildings [25]. However, for alcohol consumption, both the perception of the individual’s drinking (injunctive norm) and average drinking of each individual’s reference group (descriptive) predict alcohol consumption [29]. The same has been shown for flu vaccinations, where both injunctive messaging (whether the people closest to the parents to them think that the child should be vaccinated) and descriptive messaging (what other parents do) have an impact on parents’ decision to vaccinate their children [30]. Similar effects have been shown for front-line staff: flu vaccination uptake is impacted by both injunctive and descriptive norms [31]. In other vaccination campaigns, such as for COVID-19 vaccinations, evidence of the impact of nudges is mixed [32–34]: emphasizing the benefits of the vaccine has a positive impact on actual vaccinations, while just targeting intention to vaccinate is not enough to ensure a vaccination decision.

Given that both types of normative nudges are successful in changing decisions in the health domain, we used messaging that was injunctive and prescriptive to emphasize the individual and social benefits of the flu vaccine, keeping the messages short and to the point. This decision was recommended by the University Health Services (UHS) at our target university.

While our study was conducted prior to COVID-19, the use of these messages is consistent with the current literature on COVID-19 vaccinations, where emphasizing the benefits of the vaccines increase vaccination rates, both for individual benefits [33], social benefits (combined with different vaccine reminders) [34] and a combination of both [35]. We focus on prescriptive messages that emphasize what the individual should do (protect themselves and their community through getting the flu vaccine) and what other people do [36]. Moreover, and consistent with our study on flu vaccines for young adults, vaccine hesitancy for COVID-19 has been shown to be lower for injunctive norm treatments (social approval of a behavior compared to neutral interventions, and the use of behavioral cues for increasing vaccine acceptance are recommended for policy-making purposes [37].

Our normative messages are part of an established flu campaign at the university-level that uses posters placed in university residence halls. More specifically, flu vaccinations have also been studied when encouraged from a point of authority, such as the firm the individual is working in or the university the student attends. Types of low-cost nudge interventions such as emails done through university campuses and university health centers have been previously used to tackle vaccinations, but the effects have not been significant [38]. Implementation intentions and defaults have been used to tackle flu vaccines among employees with better results [39, 40].

For the case of vaccines, normative interventions could be used where vaccination mandates cannot be put into place for ethical reasons [41]. Vaccine normative interventions can be leveraged by highlighting aspects of the social norm and informing the public about who gets vaccinated, why they get vaccinated, and the level of protection provided by vaccines. The SAGE group on immunizations has highlighted individual and social group influence as one of the main drivers of vaccine hesitancy [42]. Therefore, positive reinforcement of vaccination social norms through nudges that highlight the individual and community effects of the vaccine have been shown to be successful in influencing vaccination decisions [43].

### Voluntary public goods provision: the case of vaccinations

From a standard economic perspective, an individual deciding to vaccinate would go through a cost-benefit analysis, where they get vaccinated if the perceived costs are lower than the perceived benefits of the vaccine [44]. An individual getting a flu vaccine does so with the aim of protecting themselves and others from the illness. In addition, an individual who gets vaccinated would generate a positive externality on other individuals, reducing the chances of contagion [5]. Vaccinations contribute to building herd immunity—protecting children, pregnant women, older adults or immunocompromised individuals and is an individual decision, and it can be interpreted as the contribution towards building herd immunity in a community [45]. Given that nobody (both vaccinated and unvaccinated) can be excluded from benefiting from herd immunity (non-excludable) and everyone receives the benefits (non-rival) from everyone’s decision to vaccinate themselves, herd immunity can be defined as a public good (For the theoretical model behind herd immunity as a public good see [45]).

Although some individuals might voluntarily seek to get a vaccination, there is an incentive to free ride and not get vaccinated [7, 11]. For example, several studies find that some parents believe that their children do not need a vaccine because other children are vaccinated [46, 47]. Other determinants of vaccine uptake found in the literature include perceptions of the social norms, beliefs about vaccine effectiveness and misperceptions of vaccine safety. Risk perception about the disease (the belief about potential harm) affects the vaccination decision, as individuals who believe they are at greater risk of getting the flu are more likely to get vaccinated [48, 49]. Perceived risk is associated with a range of anticipated emotions before getting the vaccine, such as fear, regret or worry [50, 51].

In addition, some misconceptions regarding the flu vaccine found in the literature include that it will: give people the flu, get people sick or not prevent the flu. These misconceptions are negatively associated with vaccination rates [44, 52]. Moreover, several studies focus on correcting misinformation and using norms to tackle vaccine hesitancy and observe a positive relationship between norms and vaccination decisions [53–56]. We thus incorporate individual risk attitudes and beliefs about the effectiveness and safety of the vaccine into our analysis.

## Empirical questions

Our empirical questions are inspired by the model of impure altruism [57], a special case of the joint products model [58]. Receiving a flu vaccine has both private and public benefits, as it protects the individual and helps build herd immunity. Our outcome variable, the vaccination decision, is discrete [59]. As the vaccination against the flu is recommended, the decision of whether to vaccinate or not is left to the individual.

The focus of this analysis is on testing the influence that normative messaging has on the individual decision to get a flu vaccine [13, 28]. As the two main benefits of the vaccine are protecting the individual and the individual’s community, we target the individual’s self-interest and the individual’s “community-mindedness” in the construction of the normative messages. The concept of “community-mindedness” has been used in the past to refer to the idea of helping others through priming social cohesion and affinity [60].

Therefore, we have four potential interventions depending on which characteristic of the vaccination decision is emphasized: (i) neither component of the vaccination decision is emphasized (*Baseline*), (ii) only the individual’s self-interest is emphasized (*Self)*, (iii) only the individual’s community-mindedness is emphasized (*Others)* and (iv) both messages are emphasized at the same time (*Both*). Our empirical questions are directly related to individual behavior when exposed to the messages. Our first two empirical questions focus on the overall influence of the messaging.

- Empirical Question 1: Does messaging that highlights any benefits from the vaccine (individual, community or both) have a positive influence on the decision to get vaccinated?

If normative messaging of any type is successful at increasing the likelihood of getting vaccinated compared to no messaging, we next need to know how the type of message influences the likelihood of getting vaccinated.

The decision to vaccinate will be influenced by the individual’s pro-social preferences. But individual preferences could be influenced by the strength of the norm. In the margin, individual choices could be affected by both their tendency to conform to the norm as well as how much weight the individual places on the community (“others”) versus themselves in their utility function [45]. This leads to our second empirical question.

- Empirical Question 2: Does messaging that highlights only the social benefits of the vaccine have a larger positive influence on the likelihood of vaccination compared to messaging that highlights only the individual benefits?

Messaging that emphasizes both the individual and community benefits of the vaccine together could have a stronger, weaker, or the same influence depending on the strength of both self-interest and pro-social preferences. This brings us to our third empirical question.

- Empirical Question 3: Does messaging that simultaneously highlights both the individual and social benefits of the vaccine have a larger positive influence on the decision to get vaccinated compared to messages that compare one benefit?

We conduct a field experiment to answer these empirical questions.

## Design and implementation

In this section, we discuss the assignment of participants and treatments, then describe the design of the experiment, the associated posters and the data collection process.

### Participants

The experiment was conducted during the Fall 2016 semester. We focus on undergraduate students living on campus in six different residential areas at the University of Massachusetts Amherst. Over 11,100 students were included in the study (Table 1). Participants did not know that they were part of an experiment, they only saw posters promoting the vaccination campaign, which are the standard tool used by UHS to provide information about the campaign. This reduced any potential social desirability bias [2, 61]. Posters were placed to maximize visualization within a residential area but minimize cross-contamination across treatments.

**Table 1:** Intention to treat. Number of individuals per treatment

	Baseline	Self	Others	Both	Total
Total	2197	2232	4049	2648	11126
Female	1129	975	1975	1229	5308
Male	1068	1257	2074	1419	5818

### Design

The study was conducted in conjunction with the UHS vaccination campaign. Four different informational posters were used, which differed in the normative message and the flu fact included. The posters are designed to meet the MacDonald, World Health Organization and SAGE working group recommendations to reduce vaccine hesitancy [42]. The recommendations are summarized in the 3C model and include *confidence* (trust in the effectiveness and safety of vaccines, the system that delivers them and the health professionals behind it), *complacency* (where the perceived risks of vaccine are low, thus vaccination is deemed not necessary) and *convenience* (including physical availability of vaccines, affordability and geographical accessibility). We follow *confidence* as the campaign has been consistently and successfully ran over the years with trusted professionals who have been the face of UHS as well as nurses and nursing students from the university itself. We also follow *complacency*, as the campaign and the messages target the need to protect oneself and the community, and *convenience*, as the posters highlight that most flu clinics happen through the semester in different places on campus and that vaccines are available and covered by insurance. The posters and the campaign itself is described in more detail below.

The poster campaign, which includes our nudging prompt is consistent with evidence that young adults prefer flu vaccination campaigns that rely on (1) quality and balanced information from (2) credible information sources, positioned in the (3) relevant health contexts, (4) emphasize actionable messages, and incorporate (5) persuasive campaign design [62]. Our experiment can be defined as a natural field experiment, as subjects are not informed that they are in an experiment (the campaign is part of University Health Services), the stimuli the students face their own context and environment in which they live and make decisions, such as their residential halls, study and work schedules as well as the University campus (the “real world”) [63].

The *Baseline* posters only included common information about the time and place of the flu vaccination clinics but did not include normative messages or flu facts, aiming to be a neutral poster.

The intervention posters include the information in the *Baseline* poster and add a normative message and a flu fact. The normative message was positioned prominently in the poster. The normative messages used in this experiment are injunctive and prescriptive. That is, they explicitly encourage an action to protect the subject and/or other individuals in the community from the flu [13].

The nudge-related prompt was designed to help modify risk perception about the flu and the flu vaccine, using short and concise messaging (but not focusing on clarifying vaccine misconceptions) [44, 50, 52]. The *Self* poster included the phrase “Protect yourself!”, the *Others* poster the phrase “Protect the UMass community!” and the *Both* poster the phrase “Protect yourself and the UMass community!”. Thus, the *Both* poster includes both *Self* and *Others* messages together. In the case of *Others* and *Both* posters, we present the social dilemma in a language that states what would be desirable in terms of social norms for cooperation [64].

In the design of the intervention, information on the vaccination clinics, always provided by UHS in the previous years, needed to be communicated. This limited the space available to run the three treatment arms. We used prominent placement and a font that was large relative to the administrative and clinical information to maximize the saliency of the treatment, given the space constraint. Note that similar constraints will likely exist for other vaccination or testing clinics due to the amount of information that UHS needs to communicate [65, 66].

Given the space limitations imposed upon us, our treatments could be considered relatively weak, providing potential lower bounds on the influence on the likelihood of getting vaccinated.

**Fig. 1:**
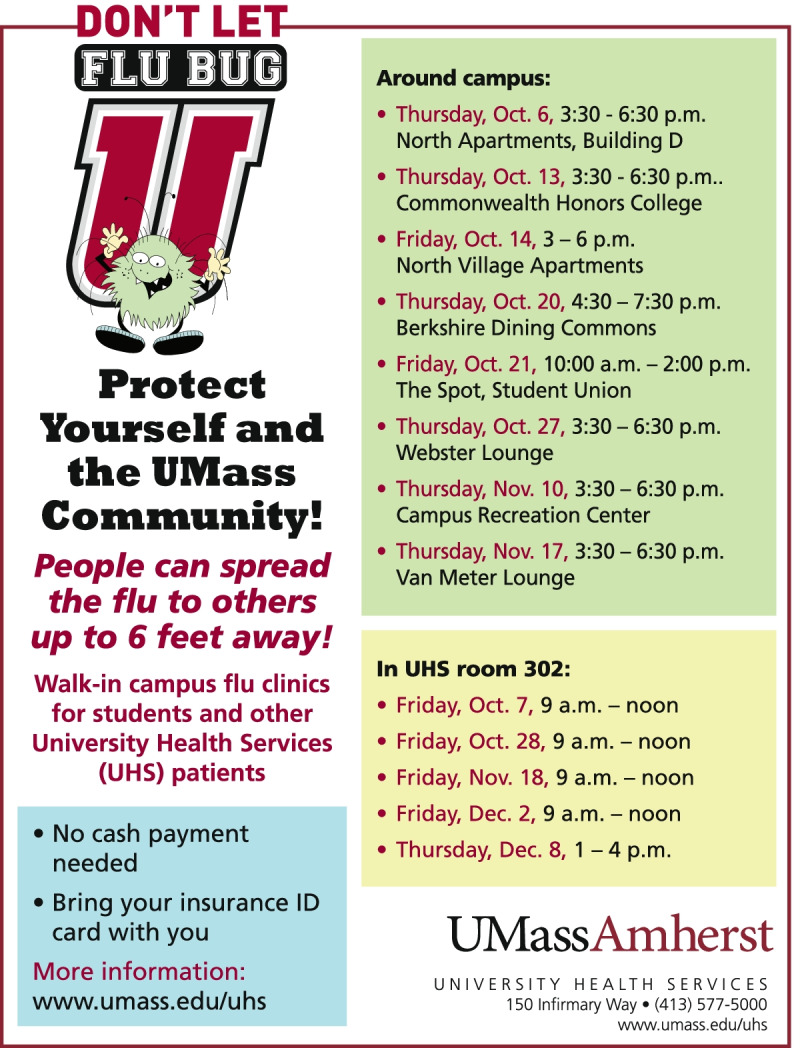
Poster for Both treatment

The flu fact is positioned below the normative message. The fact is informational and emphasizes the same information in all treatments (that a person can catch the flu from someone up to six feet away from them). It is worded to complement the normative message in terms of the protection (in terms of risk reduction) that the vaccine offers. An example of the poster for the *Both* treatment is in Fig. 1. The *Baseline, Self* and *Others* posters are shown in Additional file 1.

**Fig. 2:**
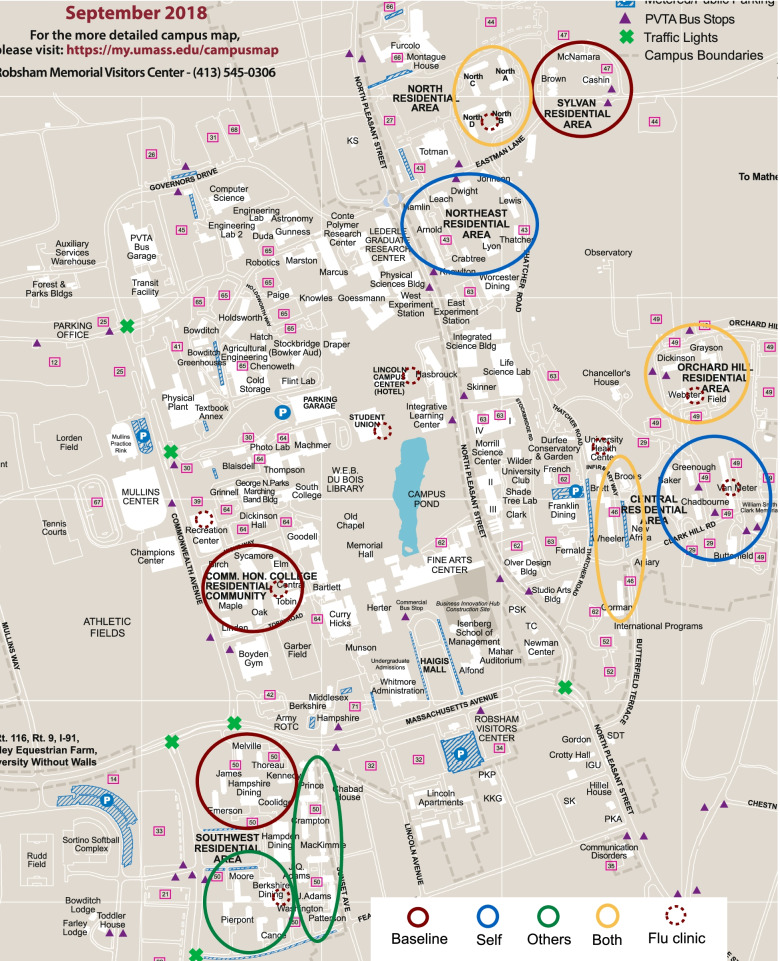
Distribution of treatments and flu clinics among residential halls on campus

Whenever possible, a complete residential area was assigned a single treatment. This was possible for four of the six residential areas. One residential area, Southwest, due to the number of students it houses, was split into two using accessibility to two dining halls (Hampshire and Berkshire Dining, see Fig. 2, bottom left for Southwest residential area). The Central residential area was naturally split into two separate areas, downhill and uphill (see Fig. 2, center-right for Central residential area).

The treatments were randomly assigned to each group of residence halls, with the aim of avoiding as much contamination between residence halls and areas as possible. We further confirmed with UHS that treatments and dates were not geographically crowded on specific weeks: that is, no two flu clinics geographically were tied to the same treatment in the same week and no two flu clinics were in the same geographical area of the campus in the same week. Further, we ensured that students residing on campus had access to a variety of locations to get vaccinated, including locations in the center of campus and UHS main clinic.

In addition, we confirmed with University Residential Services that the assignment of the majority students into residences and residential areas is random. This means that there should not be any unobserved characteristics varying by building. The location of each treatment among the residential areas on campus is specified in Fig. 2. For this reason, there are not equal numbers of students intent to treat per treatment (see Table 1).

Each treatment corresponded to a set of contiguous buildings, and each floor and entrance board had the same poster. Posters were placed in common areas of each residential hall floor and were located to maximize visibility by residents of the halls, as common areas are usually close to stairs elevators and kitchens (as per standard practice of all of UHS posters). The positioning of the posters is in line with norm saliency: the norm should be made salient as close in time as possible with the time of the decision [18]. Posters were placed in the residences the week before the flu clinics started and were checked weekly to make sure they were not (and should not be) taken down, as they are part of a University information campaign. Regarding potential contamination, entrance to residence halls by students who do not live there is subject to strict guidelines, with specific times of entrance as well as identification procedures. If contamination occurs and it is significant, it will bias the results against finding that the messages influenced vaccination decisions*. Baseline* posters and table tents were also used in common areas of the University, such as the campus center dining area, to avoid contamination between messages. A mass email to the whole University population (staff, students who live on and off-campus) was sent the day before the first clinic, with a reminder of the dates and times of the clinics and insurance information, similar to the *Baseline* information. Therefore, everyone got the same exposure to the information but differential treatment exposure depending on which poster they were assigned to.

### Flu clinics and data collection

UHS held drop-in flu clinics during the Fall 2016 semester. All flu clinics were held by UHS and were located around campus during the semester, allowing students and staff to get vaccinated closer to where they live, work or go to class. Clinics spanned from September to December 2016. Most clinics occurred before Thanksgiving break and avoided holidays and long weekends (see Additional file 1 for the dates and type of flu clinic). The times and locations of the clinics were determined by UHS, this decision being exogenous to the experimenters. Clinics were held in different areas of the University, such as dining commons, residential areas and the UHS building. This allows the University to reach as many people as possible (see Fig. 2 for the location of each clinic around campus). During the Fall, there is no other flu vaccination campaign other than the UHS campaign being rolled out on campus. Flu clinics were located in accessible areas, such as lobbies and ground floor meeting rooms. Location signs were put up outside the buildings the day of the clinics to direct students.

Students receive the vaccine at no cost as flu vaccines are covered by most insurance plans.^2^ The State of Massachusetts requires all college students to have comprehensive health insurance. For this University, students taking five or more credits are automatically enrolled in a Student Health Benefit Plan (SHBP) each semester and charged on the semester’s tuition bill or they can waive the SHBP against private health insurance. Vaccinations are billed towards the student’s health insurance and this information is already associated with their student ID in the University database, as it is a requirement.

Once students arrive to the flu clinic, they were greeted by one of the researchers or research assistants of the project and given a clipboard with the insurance forms and a survey. The survey was collected to gather individual data which we could not collect from UHS, given the confidentiality protocols at the University. The survey was voluntary for the students to fill while waiting to get vaccinated. We used a script with neutral language. The 5-minute survey was paper-based and included informed consent plus questions on health, socio-demographics, beliefs about the vaccine and its effectiveness as well as social preferences. The survey is independent from the flu vaccination campaign (the field experiment), and we clarified in the consent form that it was part of a study on healthy behaviors, not a flu vaccination study. We focused on collecting data from regular undergraduate students at the University.^3^

In addition to the surveys at the flu clinics, we also surveyed students who decided not to get a flu vaccine during the semester with an online survey. All students in the residence halls were mailed invitations to complete the survey through the University’s internal mailing procedures by mid-October. We incentivized completing the surveys by raffling two $100 Amazon gift cards. Once the surveys and the flu clinics were completed for the semester, we discarded the online surveys of those students who went to the flu clinics and filled the online survey.

## Results

We begin by examining vaccination turnout by treatment. Next, we discuss the influence of each treatment on the likelihood of getting the flu vaccine. We focus on the Fall 2016 data, unless otherwise specified. Statistical tests of balance across treatments are presented in the Additional file 1 to cross check if the assumption of independence of potential outcomes across treatments holds. Overall, and consistent with the random assignment of students to residence halls, we find that for our vaccinated participants tests are balanced for our relevant variables across treatments. For our pooled sample, we find balance across most but not all variables: to account for this lack of balance, we detail below the covariates we introduce in our analysis. More detail is provided in the Additional file 1, including balance tests for our vaccinated + unvaccinated sample.

### Vaccination turnout by treatment

The highest social benefit from vaccination occurs when herd immunity is reached. The vaccination rate in our sample is small, with 5.3% of the total intent to treat students getting vaccinated (590 undergraduate students of the 11 thousand intent to treat). The vaccination rate is significantly below what the CDC reports for the 18–24-year-old cohort, which is almost 30% [67], but it is slightly lower than other studies, reporting a rate of 7.2–7.9% [68]. For our target community, although individuals who receive the vaccine are protected against the flu, the vaccination level is low. This means that students who are at risk during the start and take up of the flu season were not protected.

In addition, it is unlikely that students are getting vaccines anywhere else during the academic semester. Although during the Fall 2016 wave we did not account for alternative vaccination locations outside the University, survey data from Fall 2017 indicates that only 7.5% of the total vaccinated surveyed students received the vaccine from somewhere other than the University flu campaign. Thus, vaccination turnout numbers are likely an accurate representation of the vaccinated undergraduate students on campus for the Fall semester. Another reason for the low vaccination turnout for the Fall semester is that students could be receiving the flu vaccine during the Winter period, when they return home. This could potentially bring our vaccination rates closer to the CDC average. However, the recommendation is to get vaccinated before the flu activity begins. Specifically, the CDC recommends receiving the flu vaccine by the end of October if possible, as it takes two weeks for the body’s immune response to fully respond [69]. In the case students are receiving the vaccine during the Winter period, the decision is not influenced by our treatments and could be a family-based decision. In addition, receiving the vaccine after the Fall term ends leaves students fully exposed to the virus during the take-up of flu activity (November to January).

**Fig. 3:**
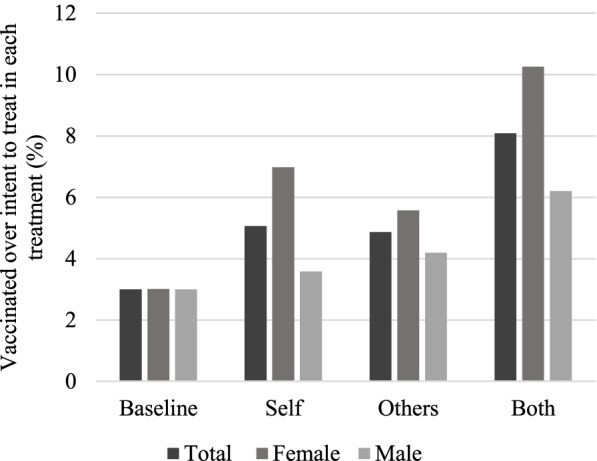
Proportion of vaccinated by treatment and gender

Compared to the *Baseline,* all treatments are significantly different in terms of the proportion of vaccinated students (proportion tests: *Self* = *Baseline*, *p* < 0.01; *Others* = *Baseline*, p < 0.01; *Both* = *Baseline*, p < 0.01). Comparing the different treatments in terms of probability of vaccination (number of vaccinated students over intent to treat in each treatment, Fig. 3), the *Both* treatment achieves a higher response to the treatment. These percentages are also different from each other (proportion tests: *Self* = *Others*, p < 0.01; *Self* = *Both,* p < 0.01; *Others* = *Both*, p < 0.01). Thus, highlighting the vaccination’s social and individual benefit together helps the most in incentivizing students’ decision to receive the vaccine at a flu clinic during the semester.

The prior literature on vaccination and gender for general populations shows that a higher percentage of females seem to accept and get vaccinated compared to males [70, 71]. If this is not the case, females tend to give more thought-out arguments (such as expressing hesitancy openly) for whether they want to get the vaccine or not [72]. Given that there could be a gender gap in our experiment, we test for these differences. In our case, and consistent with the prior work on vaccination and gender, a higher percentage of females seem to be getting vaccinated compared to the baseline regardless of the treatment (Fig. 3, Proportion tests: *Baseline* = *Self*, *p* < 0.001; *Baseline* = *Others*, *p* = 0.001; *Baseline* = *Both*, p < 0.001). For men however, the increase in vaccinations happens only for the *Both* treatment, which incorporates both the individual and community benefits of the vaccine, while for the *Others* treatment, the association is marginally significant (Proportion tests: *Baseline* = *Self*, *p* = 0.433; *Baseline* = *Others*, *p* = 0.095; *Baseline* = *Both*, p < 0.001). Thus, initial evidence suggests that our empirical question 1, which stated that any message increases vaccinations holds for females but not for males.

One explanation for the differential influence of the normative messages depending on gender could be related to risk perceptions. Prior literature argues that women could be more sensitive to social cues than men. An alternative explanation for this result is related to health care usage [73]. Women usually report a greater use of health care services, worse levels of self-perceived health as well as greater levels of morbidity and disability [74, 75].

### Likelihood of getting a flu vaccine

We next analyze the influence of the normative messaging intervention and individual characteristics on the likelihood of getting a flu vaccine. A total of 828 undergraduate students participated in this study, 360 getting the flu vaccine in the clinics and 468 not getting the vaccine. Characteristics for both sub-populations are reported in Table 2. Vaccinated and non-vaccinated students are on average similar in terms of gender (means test, *p* = 0.82) and age (means test, *p* = 0.13) and race (means test, *p* = 0.16). Students who decided to receive the vaccine self-report overall better health status (means test, *p* < 0.001). However, the question might be biased as those who get vaccinated respond to the question when arriving to get the vaccine.

**Table 2:** Descriptive statistics of survey respondents, vaccinated versus non-vaccinated

	*Vaccinated*	*Not Vaccinated*
N	360	468
Age (mean, in years)	19.23	19.4
Female (%)	55.27	54.48
Race (%)		
*White Non-Hispanic*	76.97	69.66
*African American*	2.25	4.06
*Asian*	17.42	21.15
*Other*	3.37	5.13
*Hispanic*	6.94	4.49
Self-reported health (%)		
*Good or very good*	88.86	74.79
*Satisfactory*	9.19	22.22
*Poor or bad*	1.95	2.99
Illness prevalence (%)		
*Asthma*	15.83	14.53
*Migraine*	3.89	8.97
*Depression*	8.06	10.04
*No illness*	65.38	60.9
Risk self-reported (mean, sd)	6.5 (1.7)	6.2 (1.8)
Altruism (mean, sd)	8.0 (1.6)	7.7 (1.9)
Flu effective? (mean, sd)	4.2 (0.5)	3.8 (0.8)
Flu safe? (mean, sd)	4.0 (0.8)	3.7 (1.0)
Vaccinated previously (%)	96.4	80.1

*Notes:* Self-reported risk is the answer to “Are you generally a person who is fully prepared to take risks or do you try to avoid taking risks? Please choose a value on the scale”, where value 1 means ‘not at all willing to take risks’ and the value 10 means ‘very willing to take risks’. Altruism is the answer to “How willing are you to give to good causes without expecting anything in return?”, where 1 is very unwilling to do so and 10 is very willing to do so. Flu effective? is a 5-point Likert answer to “How effective do you think the flu vaccine is?”, where 1 is very ineffective, 5 is very effective. Flu safe is a 5-point Likert answer to “How likely do you think you are to get a bad reaction from the flu vaccine?”, where 1 is very likely, 5 is very unlikely. Vaccinated previously is equal to 1 if the respondent has gotten a flu vaccine in the past, 0 otherwise

The results of logit specifications for the full sample of vaccinated and non-vaccinated students are reported in Table 3. We define our dependent variable as equal to 1 if the subject received a vaccine in a flu clinic and 0 otherwise. To be consistent with the way the sample is selected and treated (as our aim is to understand which poster yields a higher vaccination turnout), errors in each specification are clustered by treatment (as our poster assignment is based on building groups) [76, 77] and bootstrapped with 10 thousand replications. In addition, given that the number of treatments (clusters) is small and to provide asymptotic refinement, we provide the results of score-based wild cluster bootstrap tests for our regressions [78, 79]. Regression results are found in Additional file 1, including the *p*-values from the score wild cluster bootstrap. Table 3 shows the marginal percentage changes (from a linear probability model) for the relationship between the normative message treatments and receiving a flu vaccine. Other specifications can be found in Additional file 1. We have five econometric specifications. The marginal percentage changes are interpreted as the percentage point change in the likelihood of an individual getting a flu vaccine. Table 4 presents the probability of getting vaccinated under different econometric specifications.

**Table 3:** Influence of treatment on likelihood of getting a flu vaccine (marginal percentage change, linear probability model)

	(1)	(2)	(3)	(4)	(5)
(b) Assigned to *Self* treatment					
Self	0.08	0.08	0.09	0.09	0.01
	(0.05)	(0.05)	(0.06)	(0.06)	(0.08)
(c) Assigned to *Others* treatment					
Others	0.12**	0.11**	0.11**	0.12**	0.12
	(0.06)	(0.06)	(0.06)	(0.06)	(0.09)
(d) Assigned to *Both* treatment					
Both	0.20***	0.20***	0.20***	0.19***	0.19**
	(0.05)	(0.05)	(0.05)	(0.05)	(0.08)
*Controlling for:*					
Socio-demographic	No	Yes	Yes	Yes	Yes
Preferences	No	No	Yes	Yes	Yes
Beliefs	No	No	No	Yes	No
Prior vaccination	No	No	No	Yes	No
*Heterogeneity:*					
Gender interactions	No	No	No	No	Yes
*N*	*828*	*828*	*828*	*828*	*828*
χ^2^ *(p)*					
Self = Others	0.69 (0.407)	0.47 (0.493)	0.17 (0.682)	0.39 (0.532)	2.36 (0.124)
Self = Both	6.75 (0.009)	6.64 (0.009)	6.03 (0.014)	4.50 (0.034)	7.73 (0.005)
Others = Both	2.71 (0.100)	3.13 (0.077)	3.83 (0.050)	1.83 (0.176)	0.90 (0.343)
Self = Others = Both	7.37 (0.025)	7.49 (0.024)	7.45 (0.024)	4.84 (0.088)	7.73 (0.021)
Both = 2*Self	0.09 (0.758)	0.11 (0.739)	0.03 (0.863)	0.00 (0.970)	1.80 (0.179)
Both = 2*Others	0.42 (0.515)	0.21 (0.648)	0.09 (0.760)	0.46 (0.496)	0.18 (0.672)
Both≥Self+Others	0.04 (0.421)	0.01 (0.471)	0.01 (0.469)	0.16 (0.352)	0.29 (0.701)

*Notes:* *p = 0.10 **p = 0.05 ***p = 0.01*.* Dependent variable is 1 for the subject getting a vaccine, 0 for not. Marginal probabilities are reported (percentage points) only for a linear probability model where treatment variables are toggled for each case reported in the table and controls are taken at their means. Standard deviation between parentheses. Errors of the regression are clustered by treatment and bootstrapped with 10 thousand replications. Socio-demographic controls: Female, White Non-Hispanic, Illness. Preferences controls: Risk (self-reported), Altruism. Beliefs controls: Flu effective? Flu safe? Prior vaccination controls: Vaccinated Previously. Gender interactions: Self and Female, Others and Female, Both and Female. Full regression results, including the score wild cluster bootstrap tests can be found in Additional file 1

**Table 4:** Measures of prediction

**Probability of getting vaccinated (%)**					
	**(1)**	**(2)**	**(3)**	**(4)**	**(5)**
Baseline (no message)	31	31	31	29	33
Self	39	39	40	38	34
Others	44	43	42	41	45
Both	51	51	51	48	52
**Receiver operating characteristic (ROC) analysis: Area Under the Curve value**					
Cross-validated mean AUC	0.59	0.57	0.59	0.69	0.59
Standard Deviation	0.04	0.03	0.04	0.06	0.03
Bootstrap bias-corrected CI					
Lower bound	0.49	0.52	0.54	0.63	0.54
Upper bound	0.59	0.60	0.62	0.71	0.62
*Controlling for:*					
Socio-demographic	No	Yes	Yes	Yes	Yes
Preferences	No	No	Yes	Yes	Yes
Beliefs	No	No	No	Yes	No
Prior vaccination	No	No	No	Yes	No
*Heterogeneity:*					
Gender interactions	No	No	No	No	Yes
* N*	*828*	*828*	*828*	*828*	*828*

*Notes:* *p = 0.10 **p = 0.05 ***p = 0.01. The probability of getting vaccinated is the predicted probability of getting the flu vaccine if everyone was assigned to a specific treatment, under a linear probability model. The Receiver operating characteristic (ROC) analysis: Area Under the Curve value is used for comparing predictive models in both model selection and model evaluation after fitting a logit regression, and it is widely used in health-based research. It ranges from 0.5 to 1, where 1 is perfect accuracy. Acceptable predictive values start around 0.65. The Area Under the Curve was calculated using the Stata command cvauroc [80]. We use a 10 K-fold cross-validation

Our first empirical question asks whether messaging emphasizing the benefits of the vaccine positively affects the likelihood of vaccination. The second and third empirical questions compare normative treatments. If the individual and community benefits of the vaccine are complements, (see [45] for an associated model on crowding of the benefits of the vaccine), we expect that when both benefits of the vaccine are highlighted together, the final value of the likelihood of getting vaccinated compared to highlighting only one of the benefits might depend on whether the messages crowd-in or -out. We discuss this briefly below.

To answer these questions, we include dummy variables associated to the normative messages highlighted in the experiment. The variables of primary interest are *Self*, *Others* and *Both*, which are binary variables that correspond to whether the subject was assigned to a specific poster. Our *Baseline* poster had no message and no flu fact.

The estimated marginal percentage change of the normative messaging treatments is significant for the *Others* treatment and for the *Both* treatment. Compared to the *Baseline* (no message), being exposed to the messages that emphasize only the social benefit of the vaccine increases the likelihood of getting a flu vaccine by 11–12 percentage points, while being exposed by the message that highlights the individual and social benefits together increases this likelihood by 19–21 percentage points (Table 3). One concern which arises is proximity-related: the UHS building which holds some of the clinics are closest to the *Self* and *Both* treatments (see Fig. 2). If proximity influenced the decision to vaccinate in combination with the availability of UHS clinics, one would expect to find the same percentage change on those two treatments regardless of the message in the posters. However, we do not find any significant change on vaccination decisions when participants are exposed to the *Self* posters.

Regarding the second question, we find that there is no significant difference between the treatments with messages that emphasize only one benefit of the vaccine (either individual or social, *Self = Others,* χ^2^ = 0.69, *p* = 0.407 for specification (1)).

Regarding our third question, we find that emphasizing both the individual and community benefits of the flu vaccine has the strongest association with the likelihood of getting vaccinated (χ^2^ = 7.37, *p* = 0.025 for specification (1)) compared to no benefits. Also, it is important to know whether using both individual and social prompts results in crowding-in or crowding-out. If they crowd-in, the benefits of both aspects (Both) should be larger than the sum of the parts (Self + Others). To test this, we test whether Both ≥ Self+Others in the Table 3 specifications. The associated *p*-values for this test suggest that we fail to reject an equal or greater effect of our vaccine benefits for all our specifications. We can thus conclude that emphasizing both aspects together is at least neutral (additive) and could potentially result a larger benefit than emphasizing them separately: crowding-in the benefits of the flu vaccine and self-interest and pro-social preferences work together to increase vaccination turnout.

Lastly, and as a way to understand the motives behind the decision to get the flu vaccine, we incorporate unincentivized measures of (general) individual preferences. We start by including a variable to account for general altruistic motives in our specifications. We assume that altruism is a continuum, and that the strength of the preference is what we look at in our analyses first. Following the prior literature [81], we expect that if altruism influences the decision to be vaccinated, we would see a positive coefficient, as altruism shifts vaccination decisions away from individual self-interest and towards community welfare. Altruism in our main regressions (incorporated as a scale from 1 to 10) is not statistically significant. However, a more detailed analysis (provided in the Additional file 1) shows that very altruistic individuals (8–10 points in the altruistic scale) are more likely to get a flu vaccine, regardless of the treatment they were assigned to. We also tested for reciprocity and selfishness with unincentivized measures but did not find consistent or significant results. Full results of these specifications are provided in the Additional file 1.

### Prediction of vaccination outcomes

In this section we briefly discuss the predictive power of our natural experiment in terms of the probability of getting vaccinated. Table 4 shows the predicted probabilities of getting vaccinated. In our sample, a participant exposed to the treatment which highlights both benefits of the vaccine has a much higher chance of getting the vaccine (around 50%) than when being exposed to a single benefit or when not being exposed to any poster (between 31 and 45% depending on the treatment and the specification). In addition, our model including beliefs and prior history of vaccination has an acceptable predictive power when including controls related to beliefs and prior vaccination decisions (using cross-validation measures of accuracy applied in medicine and social science, Table 4). Therefore, our intervention be used to further study the influence of our campaign on vaccination outcomes provided it is properly controlled by beliefs and decision-making-related variables.

## Discussion

In this section, we discuss some of the factors which should be considered when interpreting the results and compare our results with the prior literature on non-pecuniary flu vaccine experiments.

### Caveats

We have three main caveats to be considered for this experiment. First, although we do see students coming in groups to the flu clinics, we were not able to gather information to elicit peer or family history during the Fall 2016. However, there is prior evidence on the influence of peers on flu vaccination decisions, as students coordinate vaccination decisions with their friends at the college level, with friends exchanging information and shaping each other’s beliefs about the flu virus and the vaccine [68]. Family also exerts a similar influence, either by recommending the vaccine to other family members or by setting an example and getting the vaccine [82].

Second, a bad prior season in terms of flu contagion and hospitalizations could also increase the likelihood of getting vaccinated during our flu clinics (2016–2017 season). However, the prior flu seasons to our vaccination campaign were designed by the CDC as moderate or mild [83]. Therefore, based on the information about prior seasons, we would not expect external factors related to prior flu season severity to affect vaccination decisions at the beginning of the (Fall) 2016–2017 season, when our study took place.

Third, there could be response bias in our surveys, as the number of people getting vaccinated and surveyed is much higher in proportion than those who did not. However, from the survey analysis we get similar treatment results as from the vaccinated sample, and our vaccinated and non-vaccinated samples are similar in terms of socio-economic characteristics.

### Comparison with prior literature on flu vaccines

In this section, we compare our general poster campaign to other types of interventions that can operate through social incentives to change behavior. In particular, we focus on key health-based economics literature employing low-cost strategies: namely, network analysis (including peer influence) and text messaging.

Networks and peer influence have been used to increase vaccination rates. As an example, researchers have examined the impact of identifying the “best” people to spread information about vaccination clinics [84]. In this sample of Indian villages, the villages where information was spread through those nominated by the village experienced an increase of 22% in the number of vaccinated children every month compared to villages which did not use nominated leaders to spread information. While this approach is very effective in improving vaccination rates, the cost associated with assessing who the “best” people to convey information can be high and, in low-trust environments, identifying these individuals may be infeasible.

Another approach is to improve ease-of-access to clinics. In a University setting similar to ours, as the percentage of residential halls with flu clinics grows (from one third to two thirds of the halls), the vaccination rate among the student body rises by a range of 7.2–7.9 percentage points. Over 25% of this increase can be attributed to social (peer) influence impacting vaccination decisions [68]. This can be an effective strategy if current coverage is low, although it would increase staffing costs. It is also not feasible if all halls are already covered by clinics.

Another successful, low-cost strategy for improving flu vaccine take-up is to design the campaign as opt-out rather than opt-in [40, 85]. In one of the campaigns, there was an increase in the probability of a flu shot appointment when using the opt-out strategy: 45% of the opt-out participants were vaccinated, compared to 33% in the opt-in strategy [40]. It is important to highlight that this campaign was focused on University staff, indicating that it could functionally be translated to a student population. In a second study [85] done with health care workers, there was no statistically significant differences between the opt-in and opt-out interventions. However, workers in the opt-out condition were more likely to have an appointment to get a flu vaccine, which could predict the probability of getting the vaccine.

In the United States, the use of nudging prompts through text messaging has been successful in increasing flu vaccination uptake by 5%, particularly when the prompt directly assigns a flu vaccine appointment to the individual [86]. This last study is comparable to our vaccination 5.3% intent to treat uptake results in our comparatively low-cost design focused on a large population of students.

## Conclusion

In this study, we set up a field experiment to investigate the influence of normative messaging on individual decisions to get a flu vaccine. We aim at building on the literature that uses normative influence to study how individuals’ behavior could be influenced to make healthier decisions [25, 27, 28]. We incorporate normative messaging to an already established flu vaccination campaign at a University. The posters promoting the campaign had normative messages that highlighted the individual and the social benefits of the flu vaccine. Our field experiment allows us to look at actual vaccination decisions instead of the intention to receive the vaccine. Our analysis tries to answer three main questions: whether the normative messages have a positive influence on vaccination decisions, whether messages that highlight the social benefits of the vaccine have a larger positive influence than those that highlight the individual benefits.

We find that normative messaging increases the number of students getting the flu vaccine when compared to no message. A larger number of individuals getting vaccinated means a higher level of community protection, which in the end contributes to protecting populations at risk. In line with prior studies [60], we find that strategically selected messages can increase public goods provision. When the individual and community benefits of the vaccine are emphasized together, the likelihood of getting a vaccine significantly increases.

Our results suggest that normative messaging done through a low-cost campaign could be used as a non-pecuniary policy instrument for increasing the number of vaccinated individuals within a large community [87]. In our case, messaging was strategically designed to be as salient as possible in a continuous manner, exposing students to the messages every time they are in the common areas of their residential hall. Emails and email reminders have the risk of not being open but using posters in strategic areas exposed students to the messages and information repeatedly. Normative messaging might have behavioral and health consequences, as it could help modify individual behavior. A campaign highlighting healthy behaviors (getting a vaccine) and making salient the outcome of the behavior from a social point of view (protecting the individual and the community) could be an effective and low-cost strategy to promote behavior changes [25, 28].

The experiment was successfully implemented within a University community, with undergrad students being repeatedly exposed to the messages posted in each floor of a residential area. The intervention was easy to implement and a low-cost strategy to modify behaviors, but we still see a small percentage of students receiving the vaccine outside the university clinics. It could happen that students could receive the vaccine at home during the Winter period, but that falls outside the recommended timeframe to receive the flu vaccine to be fully protected before the start of the flu season. However, we found that a low percentage of students got vaccinated after Thanksgiving. Therefore, while capturing most vaccinated students in our sample, this means that more work must be done to reach herd immunity in terms of public campaigns before the flu season peaks. Potential future work could include other non-pecuniary strategies such as “days-off” for students to get vaccinated in a one-day vaccination clinic on campus right before the flu season starts. In addition, as our target is a young adult population with widespread access to social media, new and innovative ways of transmitting information are available and should be used to increase vaccination uptake. The use of emojis, for example, which is essential in communication nowadays, is gaining traction for health uses [88, 89], and there is a current discussion on the representation of vaccinations as emojis (instead of syringe or needle emojis) [90] which should be taken forward to both in-person and online vaccination nudging campaigns.

Further research on flu vaccinations at the campus level should be directed at teasing out the influence of peer and family background (family history of chronic illnesses, vaccinations and at-risk population) from the influence of normative messaging. Contamination between residence halls should be addressed further to tease the influence of visiting another residence hall and the frequency of those visits, as students could go from one residence hall to another and be exposed to different messages. In addition, given that the normative messages influenced women (compared to men), it would be necessary to come up with new strategies or even new normative messages that can have a significant influence on male vaccination rates.

More broadly, with flu seasons being difficult to predict and ranging from mild to severe, flu vaccinations are the first barrier of protection against the flu as well as against complications from the illness. A 70% of the population vaccination target for the current year [91, 92] compared to less than half of the United States population actually getting the vaccine implies that the level of coverage against the illness was low and populations at-risk could be negatively affected by the low level of community protection, below herd immunity levels. From a public policy point of view, developing strategies and campaigns to increase vaccination on a yearly basis should be a priority. These campaigns should tackle the perception of risk related to the vaccine as well as the myths and lack of education and information surrounding flu vaccination. Moreover, the protection of others who are vulnerable or at-risk is one of the key elements of vaccination. If normative messages which include pro-social messaging can be used to increase vaccination rates in a population of young, healthy and insured individuals, a natural step should be to extend low-cost but high-impact campaigns to other at-risk communities where the number of background risks is much larger (chronic health issues, job and income instability and without health insurance). This is especially relevant nowadays for example, for the case of the coronavirus disease (COVID-19), where vaccination has been extended to whole populations and younger adults have been overall more hesitant to vaccinate than other adult groups.

## Supplementary Information

**Additional file 1.** Posters, flu clinics calendar, full regression results, leaflet for online survey recruitment and questionnaires.

## Data Availability

The data (anonymized) and code to replicate this study is available upon request. Questionnaires and experiment materials are available as an additional file jointly with this manuscript.

## Abbreviations

UHS: University Health Services

## Footnotes

1 Herd immunity happens when a significant number of people in a community are protected from getting a disease because they have either had the disease or have been vaccinated [2].

2 For nursing students and students taking courses which includes visits to hospitals and laboratory students, flu vaccinations are mandatory, so they are not part of our target population. This is a total of 60 students.

3 Administrative data on the whole University student population is always preferrable in these cases, but by University regulations we were only permitted to collect individual-specific data from our survey participants.
