# Supplementary material for: An (un)healthy social dilemma: a normative messaging field experiment with flu vaccinations
Source: Health Econ Rev. 2022 Aug 2;12:41. doi: 10.1186/s13561-022-00385-9 (PMC9344251; doi:10.1186/s13561-022-00385-9)
Supplement: Supplementary file 1 — Additional file 1. Posters, flu clinics calendar, full regression results, leaflet for online survey recruitment and questionnaires. [file 13561_2022_385_MOESM1_ESM.docx]

**Online appendix**

**Appendix A: Posters for *Baseline, Self* and *Others* treatments**

**Baseline Self**

**
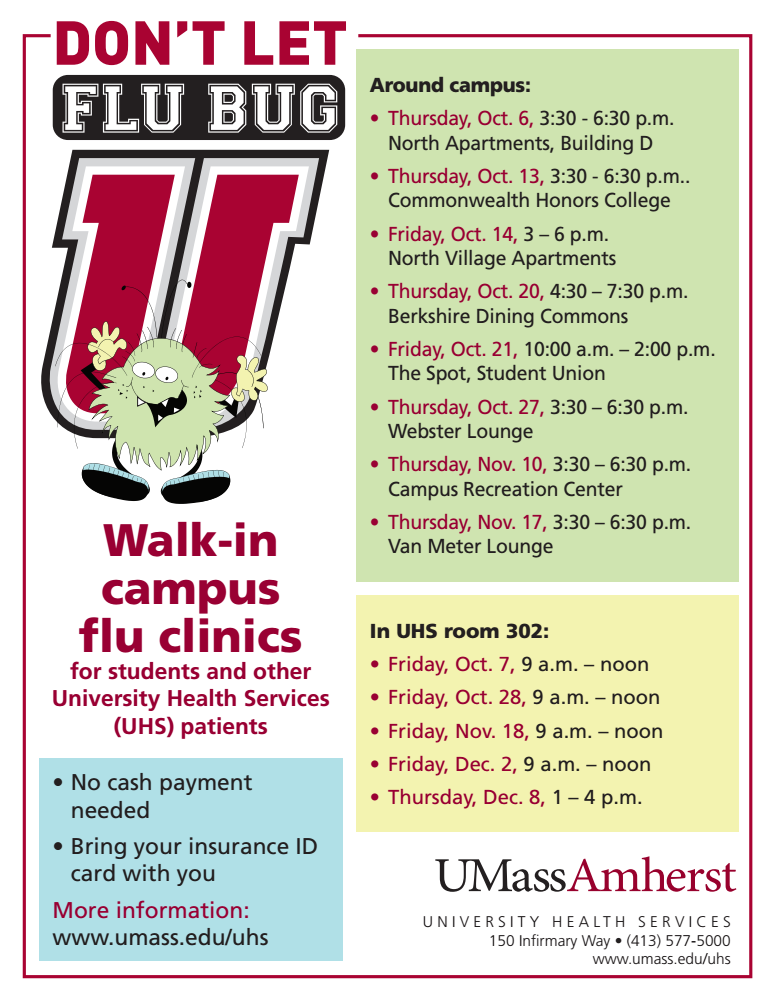

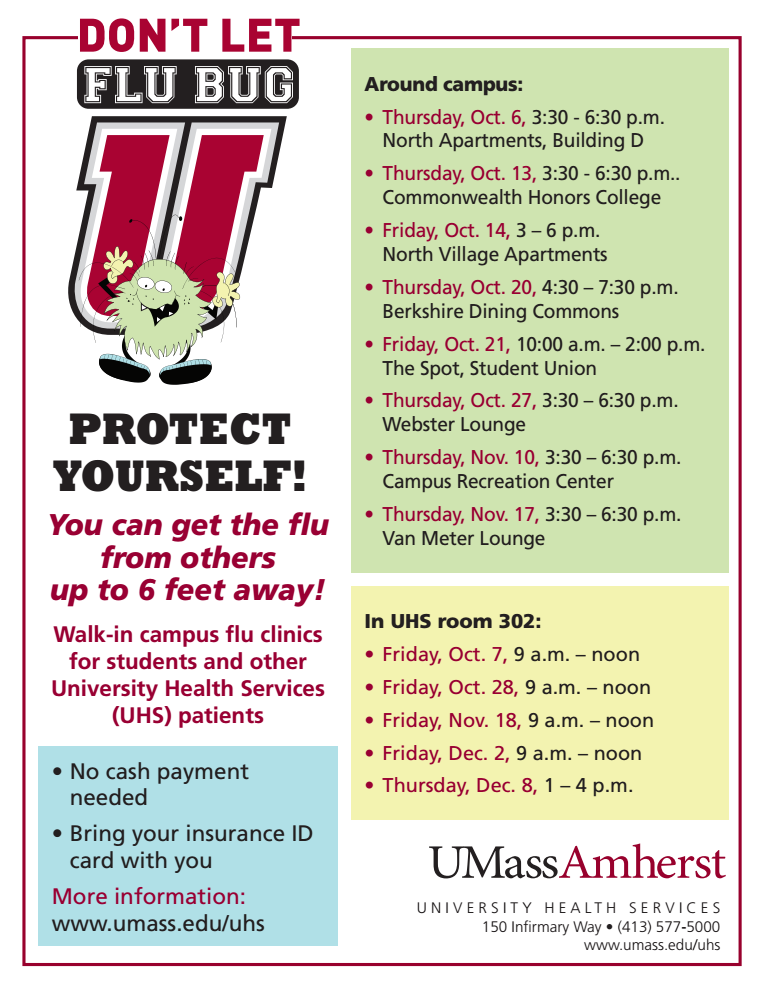
**

**Others**


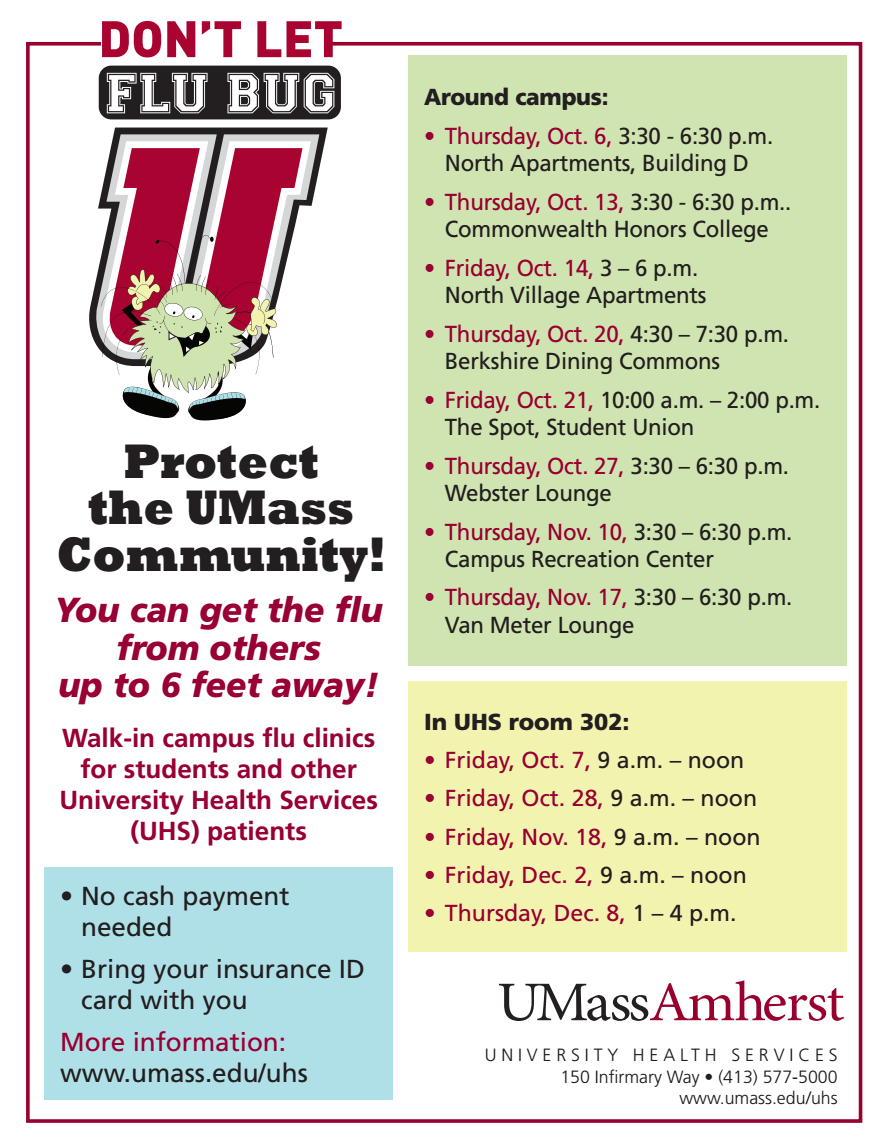


**Appendix B: Timeline of events: distribution of conditions/treatments and flu clinics during Fall 2016**

| **Month** | **Day** | **Action** |
| --- | --- | --- |
| September | 23 | Poster distribution |
|  | 26 | Closed Clinic |
|  | 27 | Closed Clinic |
|  | 28 | Email reminder to University population |
|  | 29 | Drop-in Clinic* |
|  | 30 | Closed Clinic |
| October | 6 | Closed Clinic + Drop-in Clinic* |
|  | 7 | Drop-in Clinic** |
|  | 13 | Drop-in Clinic* |
|  | 14 | Drop-in Clinic† |
|  | 20 | Drop-in Clinic* |
|  | 21 | Drop-in Clinic* |
|  | 27 | Drop-in Clinic* |
|  | 28 | Drop-in Clinic** |
|  | 31 | Closed Clinic |
| November | 8 | Closed Clinic |
|  | 10 | Drop-in Clinic* |
|  | 17 | Drop-in Clinic* |
|  | 18 | Drop-in Clinic** |
| December | 2 | Drop-in Clinic** |
|  | 8 | Drop-in Clinic** |

Notes: ** = Clinic at University Health Services, * = Clinic at Residential Area location, † = Clinic at graduate student family housing. Closed clinics were held on demand by different university groups for specific types of students who need to get the vaccine for their work or studies. We do not include in our study the responses of students taking part of the closed clinics, as for them it was either mandatory (for nursing students and laboratory students) or highly recommended by coaches (for sports teams).

**Appendix C: Statistical balance and full regression results**

**C.1. Statistical balance across treatments**

We present statistical tests of treatment balance for the socioeconomic and preference variables included in the regression analysis to cross check for independence of potential outcomes across treatments. From Table C.1., we find that there are no statistical differences in the relevant socioeconomic and preference variables between treatments for those who were vaccinated and exposed to the treatments.

For the case of the pooled sample (vaccinated and non-vaccinated), most of our variables are balanced across treatments. For the case of race, pairwise t-tests show that *Others* has a slightly higher percentage of white non-Hispanic (p-values for pairwise t-tests: *Baseline* = *Self*, p=0.97; *Baseline*=*Others,* p=0.02; *Baseline* = *Both*, p=0.52, *Self* = *Others*, p=0.01; *Self*=*Both,* p=0.50; *Others* = *Both*, p=0.03). To control for demographic differences while accounting for the minor sample imbalancedness, we include different blocks of covariates in our regressions.

*Table C.1. Balance tests for treatments, vaccinated sample (N=360)*

| Variable | Baseline | Self | Others | Both | p-value for equality of 4 group means/proportions (James' approximation) | Definition |
| --- | --- | --- | --- | --- | --- | --- |
| Female | 0.59 | 0.59 | 0.50 | 0.54 | 0.65 | 1 if the subject is female, 0 otherwise |
| White Non-Hispanic | 0.79 | 0.72 | 0.81 | 0.71 | 0.31 | 1 if the subject describes themselves as white non-Hispanic, 0 otherwise |
| Illness | 0.41 | 0.33 | 0.31 | 0.36 | 0.72 | 1 if the subject reported having at least one chronic illness, 0 if no illnesses are reported |
| Risk (self-reported) | 6.49 | 6.04 | 6.60 | 6.21 | 0.17 | Self-reported risk (unincentivized measure) is the answer to “Are you generally a person who is fully prepared to take risks or do you try to avoid taking risks? Please choose a value on the scale”, where value 1 means ‘not at all willing to take risks’ and the value 10 means ‘very willing to take risks'. |
| Altruism | 7.71 | 7.73 | 7.84 | 7.80 | 0.41 | Altruism (unincentivized measure) is the answer to “How willing are you to give to good causes without expecting anything in return?”, where 1 is very unwilling to do so and 10 is very willing to do so. |
| Flu safe | 3.79 | 3.89 | 3.81 | 3.89 | 0.67 | Flu safe is a 5-point Likert answer to “How likely do you think you are to get a bad reaction from the flu vaccine?”, where 1 is very likely, 5 is very unlikely. |
| Flu effective | 3.95 | 3.95 | 3.94 | 4.11 | 0.27 | Flu effective? is a 5-point Likert answer to “How effective do you think the flu vaccine is?”, where 1 is very ineffective, 5 is very effective. |

*Note: Vaccinated Previously (1 if previously vaccinated against influenza, 0 otherwise) is not included as testing yields a singular covariance matrix, as the majority of individuals in the vaccinated sample were vaccinated previously).*

*Table C.2. Balance tests for treatments, all sample (N=828)*

| Variable | Baseline | Self | Others | Both | p-value for equality of 4 group means/proportions (James' approximation) | Definition |
| --- | --- | --- | --- | --- | --- | --- |
| Female | 0.61 | 0.51 | 0.54 | 0.55 | 0.31 | 1 if the subject is female, 0 otherwise |
| White Non-Hispanic | 0.68 | 0.68 | 0.80 | 0.71 | 0.02 | 1 if the subject describes themselves as white non-Hispanic, 0 otherwise |
| Illness | 0.45 | 0.35 | 0.34 | 0.37 | 0.17 | 1 if the subject reported having at least one chronic illness, 0 if no illnesses are reported |
| Risk (self-reported) | 6.49 | 6.04 | 6.60 | 6.21 | 0.01 | Self-reported risk (unincentivized measure) is the answer to “Are you generally a person who is fully prepared to take risks or do you try to avoid taking risks? Please choose a value on the scale”, where value 1 means ‘not at all willing to take risks’ and the value 10 means ‘very willing to take risks'. |
| *Risk  averse* | *0.14* | *0.21* | *0.12* | *0.16* | *0.10* | *1 if subject self-reports risk level below 4 (included)* |
| Altruism | 7.71 | 7.73 | 7.84 | 7.80 | 0.87 | Altruism (unincentivized measure) is the answer to “How willing are you to give to good causes without expecting anything in return?”, where 1 is very unwilling to do so and 10 is very willing to do so. |
| Flu safe | 3.79 | 3.89 | 3.81 | 3.89 | 0.58 | Flu safe is a 5-point Likert answer to “How likely do you think you are to get a bad reaction from the flu vaccine?”, where 1 is very likely, 5 is very unlikely. |
| Flu effective | 3.95 | 3.95 | 3.94 | 4.11 | 0.02 | Flu effective? is a 5-point Likert answer to “How effective do you think the flu vaccine is?”, where 1 is very ineffective, 5 is very effective. |
| *Flu very effective* | *0.20* | *0.24* | *0.21* | *0.28* | *0.15* | *1 if subject answers that the vaccine is very effective (5)* |
| Vaccinated previously | 0.86 | 0.86 | 0.88 | 0.88 | 0.87 | 1 if the subject has gotten a flu vaccine in the past, 0 otherwise. |

**C.2. Full regression results**

Our analysis includes five blocks of controls: socio-economic, preferences, beliefs, prior vaccination and gender interactions. The first econometric specification only includes dummies for the three treatments (*Baseline* is omitted). The second specification introduces socio-economic controls to understand how individual characteristics could influence the decision to get vaccinated. These include gender (*Female* is equal to 1 if the subject is female, 0 otherwise), white non-Hispanic (equal to 1 if the subject describes themselves as white non-Hispanic, 0 otherwise), and illness (equal to 1 if the subject reported having at least one chronic illness, 0 if no illnesses are reported).

A third specification incorporates self-reported preferences. As a proxy for individual risk attitudes, we include *Risk,* a self-reported measure of the subjects’ degree of risk aversion. *Risk* responds to the hypothetical question “How do you see yourself: are you generally a person who is fully prepared to take risks or do you try to avoid taking risks?”. In this case, 1 means ‘not at all willing to take risks’ and 10 means ‘very willing to take risks'. We also introduce a self-reported measure of altruism. We asked students “How willing are you to give to good causes without expecting anything in return?”, where 1 is very unwilling to do so and 10 is very willing to do so.

The fourth specification includes individual beliefs regarding the safety and effectiveness of the flu vaccine and a question on prior vaccination. *Flu Effective?* is based on the question “How effective do you think the flu vaccine is?”, where 1 is being very ineffective and 5 very effective. *Flu Safe?* is based on the question “How likely do you think you are to get a bad reaction from the flu vaccine?” where 1 is very unlikely and 5 very likely.

One of the reasons why someone would think that the flu vaccine is “safe” could be related to their history of flu vaccinations. It is more likely that an individual gets the vaccine during the current flu vaccination campaign if they have gotten the flu vaccine in prior flu seasons. Several studies have shown that past behavior is a strong predictor of flu vaccine acceptance (Schmid et al. 2017). For this reason, the history of prior vaccination could be a relevant explanatory variable for the decision to get vaccinated. We include the variable *Vaccinated Previously*, with value 1 if the student responds positively to whether he/she has gotten a flu shot before.

Our final specification includes with interactions between treatment and gender, to be consistent with the vaccination turnout findings.

*Table C.3. Influence of treatment on likelihood of getting a flu vaccine (with controls, logit specification)*

|  | (1) | (2) | (3) | (4) | (5) |
| --- | --- | --- | --- | --- | --- |
| Self | 0.36 | 0.35 | 0.40 | 0.40 | 0.02 |
|  | (0.24) | (0.24) | (0.24) | (0.25) | (0.38) |
| Others | 0.54** | 0.49** | 0.48** | 0.54** | 0.52 |
|  | (0.24) | (0.25) | (0.25) | (0.26) | (0.38) |
| Both | 0.84*** | 0.83*** | 0.86*** | 0.82*** | 0.79** |
|  | (0.22) | (0.22) | (0.22) | (0.24) | (0.35) |
| Female |  | 0.05 | 0.60 | 0.17 | -0.12 |
|  |  | (0.15) | (0.15) | (0.16) | (0.39) |
| White Non-Hispanic |  | 0.22 | 0.18 | 0.11 | 0.19 |
|  |  | (0.16) | (0.16) | (0.17) | (0.16) |
| Illness |  | -0.18 | -0.18 | -0.24 | -0.17 |
|  |  | (0.15) | (0.15) | (0.16) | (0.15) |
| Risk (self-reported) |  |  | 0.09** | 0.09* | 0.10** |
|  |  |  | (0.04) | (0.05) | (0.04) |
| Altruism |  |  | 0.08* | 0.07 | 0.07* |
|  |  |  | (0.04) | (0.05) | (0.04) |
| Flu effective? |  |  |  | 0.62*** |  |
|  |  |  |  | (0.13) |  |
| Flu safe? |  |  |  | 0.12 |  |
|  |  |  |  | (0.09) |  |
| Vaccinated previously |  |  |  | 1.50*** |  |
|  |  |  |  | (0.35) |  |
| Self * Female |  |  |  |  | 0.70 |
|  |  |  |  |  | (0.50) |
| Others * Female |  |  |  |  | -0.09 |
|  |  |  |  |  | (0.50) |
| Both * Female |  |  |  |  | 0.11 |
|  |  |  |  |  | (0.46) |
| Constant | -0.79*** | -0.90*** | -2.11*** | -6.28*** | -2.00*** |
|  | (0.19) | (0.24) | (0.45) | (0.43) | (0.51) |
| *N* | *828* | *828* | *828* | *828* | *828* |
| *Wald Chi^2^* | *16.72* | *19.84* | *30.15* | *91.9* | *34.15* |
| *Log likelihood* | *-558.103* | *-556.419* | *-551.052* | *-507.256* | *-549.063* |
| *Pseudo R^2^* | *0.015* | *0.018* | *0.000* | *0.105* | *0.031* |

*Notes: * p=0.10 ** p=0.05 *** p=0.01 for the score wild cluster bootstrap tests (MacKinnon et al. 2018). Dependent variable is 1 for the subject getting a vaccine, 0 for not. Standard deviation between parentheses. Errors of the regression are clustered by treatment and bootstrapped with 10 thousand replications. Presented in the table are the coefficients of the logit specification used in the main manuscript to perform our calculations (Tables 3 and 4).*

*Table C.4. Influence of individual preferences on likelihood of getting a flu vaccine (logit specification)*

|  | Altruism (dichotomous) | | | | Reciprocity (dichotomous) | | | Selfish (dichotomous) | | |
| --- | --- | --- | --- | --- | --- | --- | --- | --- | --- | --- |
|  | (1.a) | (1.b) | (1.c) | (1.d) | (2.a) | (2.b) | (2.c) | (3.a.) | (3.b.) | (3.c.) |
| Self | 0.38* | 0.39* | 0.01 | 0.43 | 0.42* | 0.42* | 0.05 | 0.38* | 0.40* | 0.00 |
|  | (0.24) | (0.25) | (0.38) | (0.46) | (0.24) | (0.25) | (0.38) | (0.24) | (0.26) | (0.38) |
| Others | 0.49** | 0.54** | 0.53 | 0.63 | 0.50** | 0.50** | 0.56 | 0.47** | 0.54** | 0.49 |
|  | (0.25) | (0.26) | (0.38) | (0.45) | (0.25) | (0.26) | (0.38) | (0.25) | (0.26) | (0.38) |
| Both | 0.86*** | 0.82*** | 0.80** | 1.41** | 0.87*** | 0.83*** | 0.83*** | 0.83*** | 0.80*** | 0.78** |
|  | (0.22) | (0.24) | (0.35) | (0.41) | (0.22) | (0.24) | (0.35) | (0.22) | (0.24) | (0.35) |
| Female | 0.04 | 0.16 | -0.14 | 0.04 | 0.09 | 0.20 | -0.06 | 0.10 | 0.21 | -0.07 |
|  | (0.16) | (0.16) | (0.39) | (0.15) | (0.15) | (0.16) | (0.39) | (0.15) | (0.16) | (0.39) |
| White Non-Hispanic | 0.16 | 0.10 | 0.18 | 0.14 | 0.19 | 0.13 | 0.21 | 0.19 | 0.13 | 0.21 |
|  | (0.16) | (0.17) | (0.16) | (0.16) | (0.16) | (0.17) | (0.16) | (0.16) | (0.17) | (0.16) |
| Illness | -0.20 | -0.25 | -0.19 | -0.20 | -0.17 | -0.23 | -0.17 | -0.18 | -0.24 | -0.18 |
|  | (0.15) | (0.16) | (0.15) | (0.15) | (0.15) | (0.16) | (0.15) | (0.15) | (0.16) | (0.15) |
| Risk (self-reported) | 0.09** | 0.09* | 0.10** | 0.10** | 0.10* | 0.09* | 0.11** | 0.11*** | 0.10** | 0.12*** |
|  | (0.04) | (0.05) | (0.04) | (0.04) | (0.04) | (0.04) | (0.04) | (0.04) | (0.04) | (0.04) |
| Altruistic | 0.44*** | 0.36** | 0.44*** | 0.87** |  |  |  |  |  |  |
|  | (0.15) | (0.16) | (0.15) | (0.43) |  |  |  |  |  |  |
| Reciprocal |  |  |  |  | 0.33 | 0.17 | 0.33* |  |  |  |
|  |  |  |  |  | (0.21) | (0.23) | (0.21) |  |  |  |
| Selfish |  |  |  |  |  |  |  | -0.31** | -0.23 | -0.30* |
|  |  |  |  |  |  |  |  | (0.16) | (0.17) | (0.15) |
| Flu effective? |  | 0.61*** |  |  |  | 0.61*** |  |  | 0.61*** |  |
|  |  | (0.13) |  |  |  | (0.12) |  |  | (0.13) |  |
| Flu safe? |  | 0.12 |  |  |  | 0.12 |  |  | 0.13 |  |
|  |  | (0.09) |  |  |  | (0.09) |  |  | (0.09) |  |
| Vaccinated previously |  | 1.48*** |  |  |  | 1.49*** |  |  | 1.49*** |  |
|  |  | (0.35) |  |  |  | (0.34) |  |  | (0.34) |  |
| Self * Female |  |  | 0.69 |  |  |  | 0.68 |  |  | 0.69 |
|  |  |  | (0.49) |  |  |  | (0.49) |  |  | (0.49) |
| Others * Female |  |  | -0.10 |  |  |  | -0.13 |  |  | -0.08 |
|  |  |  | (0.49) |  |  |  | (0.50) |  |  | (0.49) |
| Both * Female |  |  | 0.10 |  |  |  | 0.06 |  |  | 0.07 |
|  |  |  | (0.44) |  |  |  | (0.46) |  |  | (0.44) |
| Self * Altruistic |  |  |  | -0.07 |  |  |  |  |  |  |
|  |  |  |  | (0.55) |  |  |  |  |  |  |
| Others * Altruistic |  |  |  | -0.21 |  |  |  |  |  |  |
|  |  |  |  | (0.54) |  |  |  |  |  |  |
| Both * Altruistic |  |  |  | -0.86* |  |  |  |  |  |  |
|  |  |  |  | (0.49) |  |  |  |  |  |  |
| Constant | -1.75*** | -5.93*** | -2.00*** | -2.01*** | -1.88*** | -5.99*** | -1.82*** | -1.54*** | -5.82*** | -1.48*** |
|  | (0.37) | (0.68) | (0.51) | (0.48) | (0.40) | (0.69) | (0.46) | (0.37) | (0.69) | (0.51) |
| *N* | *828* | *828* | *828* | *828* | *828* | *828* | *828* | *828* | *828* | *828* |
| *Wald Chi^2^* | *34.95* | *94.63* | *38.34* | *35.53* | *30.02* | *92.57* | *34.62* | *29.50* | *91.73* | *34.04* |
| *Log likelihood* | *-548.449* | *-505.797* | *-546.375* | *-545.297* | *-551.487* | *-508.030* | *-549.348* | *-550.867* | *-507.371* | *-548.824* |
| *Pseudo R^2^* | *0.032* | *0.108* | *0.036* | *0.038* | *0.027* | *0.104* | *0.031* | *0.028* | *0.105* | *0.032* |
| *p-value* |  |  |  |  |  |  |  |  |  |  |
| *Both* ≥ *Self+Others* | *0.489* | *0.741* | *0.714* | *0.726* | *0.442* | *0.329* | *0.679* | *0.469* | *0.741* | *0.723* |

*Notes: * p=0.10 ** p=0.05 *** p=0.01 for the score wild cluster bootstrap tests (MacKinnon et al. 2018). Dependent variable is 1 for the subject getting a vaccine, 0 for not. Standard deviation between parentheses. Errors of the regression are clustered by treatment and bootstrapped with 10 thousand replications. . Presented in the table are the coefficients of the logit specification. Altruistic is 1 if the person responded 8-10 to the question “How willing are you to give to good causes without expecting anything in return?”, where 1 is very unwilling to do so and 10 is very willing to do so, 0 otherwise. Reciprocal is 1 if the person responded 8-10 to the question “How well does the following statement describe you? When someone does me a favor I am willing to return it” where 1 is does not describe me at all and 10 is describes me perfectly, 0 otherwise. Selfish is 1 if the person strongly agrees with the statement “These days people need to look after themselves and not overly worry about others”, 0 otherwise.*

**Appendix D: Recruitment materials, flu clinic and online surveys**

***Online survey recruitment flyer***

***Front***

**

***Back***

**

***Flu clinic questionnaire***

Consent Form

You are being invited to participate in a research study conducted by Dr. Angela C. M. de Oliveira and Ms. Irene Mussio from the Department of Resource Economics at University of Massachusetts Amherst. You were selected to participate in this study because you decided to get a flu shot. The purpose of this research study is to better understand the factors that affect healthy behaviors. If you agree to take part in this study, you will be asked to complete a short survey on the next page. This survey will ask about your socioeconomic characteristics, health status and behaviors and it will take you approximately 5 minutes to complete. You may not directly benefit from this research; however, we hope that your participation in the study may help the University of Massachusetts Amherst redesign health-related policies and incentives to engage in healthy behaviors. Your answers in this study will remain confidential.

**Your participation in this study is completely voluntary and you can withdraw at any time. You are free to skip any question you choose.**

If you have questions about this project or if you have a research-related problem, you may contact the researchers, Dr. Angela C. M. de Oliveira at [adeolive@resecon.umass.edu](mailto:adeolive@resecon.umass.edu) and Ms. Irene Mussio at [imussiogarci@resecon.umass.edu](mailto:imussiogarci@resecon.umass.edu). If you have any questions concerning your rights as a research subject, you may contact the University of Massachusetts Amherst Human Research Protection Office (HRPO) at (413) 545-3428 or humansubjects@ora.umass.edu.

By proceeding to the survey on the next page you are indicating that you are at least 18 years old, have read and understood this consent form and agree to participate in this research study. Please keep this page for your records and return the survey to the researchers. Please DO NOT write your name on the survey.

_____________________________________________________

Signature

Please take a moment to answer this brief survey. Your participation is highly appreciated. Email address will be used to send a debriefing about the study.

- 1. Your UMass email: __________________________________
  2. What is your gender? ^1^ Male ^2^ Female ^3^ I prefer not to answer
  3. What is your age? ____________
  4. Are you a: ^1^ Full time student ^2^ Part-time student ^3^ Not a student

- 1. Are you a: ^1^ Undergraduate student ^2^ Graduate student
  2. Are you living: ^0^ Off campus ^1^ On campus
  3. If on campus, which dorm? ____________________
  4. Do you work? ^1^ No ^2^ Part time job (<10 hours a week)

^5^ Part time job (10-19 hours a week) ^3^ Part time job (20-39 hours a week) ^4^ Full time job (>39 hours a week)

- 1. Select the racial category that ^1^ White ^2^ Black or African American

best describes you: ^3^ American Indian or Alaska Native ^4^ Asian

^5^ Native Hawaiian/Pacific Islander ^6^ Other _______________

- 1. What is your ethnicity? ^1^ Hispanic or Latino ^0^ Not Hispanic nor Latino

11. How would you describe your current health?

^5^ Very good ^4^ Good ^3^ Satisfactory ^2^ Poor ^1^ Bad

12. Do you have a health problem that limits you in normal everyday life?

^3^ Yes, severely ^2^ Yes, somewhat ^1^ No, not at all

13. If you answered Yes, have you had this health problem for more than a year? ^1^ Yes ^0^ No

1. How many days were you unable to go to class in the last year due to illness? _______________ days (please state the total number of days, not just the days for which you had an official note from your doctor)
2. Has a doctor ever diagnosed you to have one or more of the following illnesses?

^0^ No illness diagnosed

^1^ Sleep disorder

^2^ Diabetes

^3^ Asthma

^4^ Cardiac disease (cardiac insufficiency, weak heart)

^5^ Cancer

^6^ Stroke

^7^ Migraine

^8^ High blood pressure

^9^ Depression

^10^ Dementia

^11^ Joint diseases (including arthritis, rheumatism)

^12^ Chronic back trouble

^13^ Other illness (please specify)

1. How many hours do you sleep on average on a normal day? (give only whole hours)________ How many hours on a normal weekend? _________
2. If you live in a dorm, have you seen the flu clinic posters inside the building? ^1^ Yes ^0^ No

|  | a. Gotten a flu shot? | b. Donated blood? | c. Done physical exercise (twice a week or more)? |
| --- | --- | --- | --- |
| 1. Have you ever… | ^1^ Yes ^0^ No | ^1^ Yes ^0^ No | ^1^ Yes ^0^ No |
| 1. Why did you make this choice? |  |  |  |

|  | a. Get a flu shot? | b. Donate blood? | c. Do physical exercise (twice a week or more)? |
| --- | --- | --- | --- |
| 1. Are you planning at some point in the future to… | ^1^ Yes ^0^ No | ^1^ Yes ^0^ No | ^1^ Yes ^0^ No |

1. How effective do you think the flu vaccine is?

^5^ Very effective ^4^ Effective ^3^ Neither effective nor ineffective ^2^ Ineffective ^1^ Very Ineffective

1. How likely do you think you are to get a bad reaction from the flu vaccine?

^5^ Very likely ^4^ Likely ^3^ Neither likely nor unlikely ^2^ Unlikely ^1^ Very Unlikely

1. How likely do you think donating blood will help if you need blood?

^5^ Very likely ^4^ Likely ^3^ Neither likely nor unlikely ^2^ Unlikely ^1^ Very Unlikely

1. How much do you think exercising will help improving your health status?

^5^ Very likely ^4^ Likely ^3^ Neither likely nor unlikely ^2^ Unlikely ^1^ Very Unlikely

1. How do you see yourself: are you generally a person who is fully prepared to take risks or do you try to avoid taking risks? Please choose a value on the scale, where the value 1 means ‘not at all willing to take risks’ and the value 10 means ‘very willing to take risks'.

Not at all willing to take risks Very willing to take risks

1 2 3 4 5 6 7 8 9 10

1. Some people believe that individuals can decide their own destiny, while others think that it is impossible to escape a predetermined fate. What comes closest to your view on the scale below?

Everything is determined by fate People shape their fate themselves

1 2 3 4 5 6 7 8 9 10

1. Please tell me how much you agree with the following statement: “These days people need to look after themselves and not overly worry about others”

^5^ Strongly agree ^4^ Agree ^3^ Neither agree nor disagree ^2^ Disagree ^1^ Strongly disagree

1. How willing are you to give to good causes without expecting anything in return?

Very unwilling to do so Very willing to do so

1 2 3 4 5 6 7 8 9 10

1. How well does the following statement describe you? “When someone does me a favor I am willing to return it”

Does not describe me at all Describes me perfectly

1 2 3 4 5 6 7 8 9 10

***Online questionnaire***

Please take a moment to answer this brief survey. Your participation is highly appreciated. Email address will be used to send a debriefing about the study.

1. Your UMass email: __________________________________
2. What is your gender? ^1^ Male ^2^ Female ^3^ I prefer not to answer
3. What is your age? ____________
4. Are you a: ^1^ Full time student ^2^ Part-time student ^3^ Not a student

1. Are you a: ^1^ Undergraduate student ^2^ Graduate student
2. Are you living: ^0^ Off campus ^1^ On campus
3. If on campus, which dorm? ____________________
4. Do you work? ^1^ No ^2^ Part time job (<10 hours a week)

^5^ Part time job (10-19 hours a week) ^3^ Part time job (20-39 hours a week) ^4^ Full time job (>39 hours a week)

1. Select the racial category that ^1^ White ^2^ Black or African American

best describes you: ^3^ American Indian or Alaska Native ^4^ Asian

^5^ Native Hawaiian/Pacific Islander ^6^ Other _______________

1. What is your ethnicity? ^1^ Hispanic or Latino ^0^ Not Hispanic nor Latino

11. How would you describe your current health?

^5^ Very good ^4^ Good ^3^ Satisfactory ^2^ Poor ^1^ Bad

12. Do you have a health problem that limits you in normal everyday life?

^3^ Yes, severely ^2^ Yes, somewhat ^1^ No, not at all

13. If you answered Yes, have you had this health problem for more than a year?

^1^ Yes ^0^ No

1. How many days were you unable to go to class in the last year due to illness? _______________ days (please state the total number of days, not just the days for which you had an official note from your doctor)
2. Has a doctor ever diagnosed you to have one or more of the following illnesses?

^1^ Sleep disorder

^2^ Diabetes

^3^ Asthma

^4^ Cardiac disease (cardiac insufficiency, weak heart)

^5^ Cancer

^6^ Stroke

^7^ Migraine

^8^ High blood pressure

^9^ Depression

^10^ Dementia

^11^ Joint diseases (including arthritis, rheumatism)

^12^ Chronic back trouble

^13^ Other illness (please specify) ____________________

^14^ No illness diagnosed

1. How many hours do you sleep on average on a normal day? (give only whole hours)________ How many hours on a normal weekend? _________

|  | a. Gotten a flu shot? | b. Donated blood? | c. Done physical exercise (twice a week or more)? |
| --- | --- | --- | --- |
| 1. Have you ever… | ^1^ Yes ^0^ No | ^1^ Yes ^0^ No | ^1^ Yes ^0^ No |
| 1. Why did you decide that? (please respond why did you decide/ did not decide to do the three actions) |  |  |  |

|  | a. Get a flu shot? | b. Donate blood? | c. Do physical exercise (twice a week or more)? |
| --- | --- | --- | --- |
| 1. Are you planning at some point in the future to… | ^1^ Yes ^0^ No | ^1^ Yes ^0^ No | ^1^ Yes ^0^ No |

1. How effective do you think the flu vaccine is?

^5^ Very effective ^4^ Effective ^3^ Neither effective nor ineffective ^2^ Ineffective ^1^ Very Ineffective

1. How likely do you think you are to get a bad reaction from the flu vaccine?

^5^ Very unlikely ^4^ Unlikely ^3^ Neither likely nor unlikely ^2^ Likely ^1^ Very Likely

1. How do you see yourself: are you generally a person who is fully prepared to take risks or do you try to avoid taking risks? Please choose a value on the scale, where the value 1 means ‘not at all willing to take risks’ and the value 10 means ‘very willing to take risks'.

Not at all willing to take risks 1 2 3 4 5 6 7 8 9 10 Very willing to take risks

1. Some people believe that individuals can decide their own destiny, while others think that it is impossible to escape a predetermined fate. What comes closest to your view on the scale below?

Everything is determined by fate 1 2 3 4 5 6 7 8 9 10 People shape their fate themselves

1. Please tell me how much you agree with the following statement: “These days people need to look after themselves and not overly worry about others”

^5^ Strongly agree ^4^ Agree ^3^ Neither agree nor disagree ^2^ Disagree ^1^ Strongly disagree

1. How willing are you to give to good causes without expecting anything in return?

Very unwilling to do so 1 2 3 4 5 6 7 8 9 10 Very willing to do so

1. How well does the following statement describe you? “When someone does me a favor I am willing to return it”

Does not describe me at all 1 2 3 4 5 6 7 8 9 10 Describes me perfectly
